# Impact of Digital Engagement on Weight Loss Outcomes in Obesity Management Among Individuals Using GLP-1 and Dual GLP-1/GIP Receptor Agonist Therapy: Retrospective Cohort Service Evaluation Study

**DOI:** 10.2196/69466

**Published:** 2025-03-31

**Authors:** Hans Johnson, David Huang, Vivian Liu, Mahmoud Al Ammouri, Christopher Jacobs, Austen El-Osta

**Affiliations:** 1 Menwell Ltd (t/a Voy) London United Kingdom; 2 Department of Digital Health and Care, School of Engineering Mathematics and Technology University of Bristol Bristol United Kingdom; 3 Self-Care Academic Research Unit (SCARU), Department of Primary Care & Public Health, School of Public Health Imperial College London London United Kingdom; 4 Department of Psychology University of Bath Bath United Kingdom

**Keywords:** obesity, weight loss, semaglutide, tirzepatide, digital health, engagement, behavior, coaching, retrospective study, service evaluation

## Abstract

**Background:**

Obesity is a global public health challenge. Pharmacological interventions, such as glucagon-like peptide-1 (GLP-1) receptor agonists (eg, semaglutide) and dual GLP-1/gastric inhibitory polypeptide receptor agonists (eg, tirzepatide), have led to significant weight loss among users. Digital health platforms offering behavioral support may enhance the effectiveness of these medications.

**Objective:**

This retrospective service evaluation investigated the impact of engagement with an app-based digital weight loss program on weight loss outcomes among individuals using GLP-1 receptor agonists (semaglutide) and dual GLP-1/gastric inhibitory polypeptide receptor agonists (tirzepatide) in the United Kingdom over 5 months.

**Methods:**

Data were collected from the Voy weight loss digital health platform between February 2023 and August 2024. Participants were adults aged 18-75 years with a BMI ≥30 or ≥27.5 kg/m^2^ with the presence of obesity-related comorbidities who initiated a weight management program involving semaglutide or tirzepatide. Engagement was defined based on attendance at coaching sessions, frequency of app use, and regular weight tracking. Participants were categorized as “engaged” or “nonengaged” accordingly. Weight loss outcomes were assessed over a period of up to 5 months. Statistical analyses included chi-square tests, independent *t* tests, Kaplan-Meier survival analysis, and calculations of Cohen *d* for effect sizes.

**Results:**

A total of 57,975 participants were included in the analysis, with 31,407 (54.2%) classified as engaged and 26,568 (45.8%) as nonengaged. Engaged participants achieved significantly greater weight loss at each time point. At month 3, engaged participants had a mean weight loss of 9% (95% CI 9% to 9.1%) compared with 5.9% (95% CI 5.9% to 6%) in nonengaged participants (*P*<.001), representing a mean difference of 3.1 percentage points (95% CI 3.1% to 3.1%). A Cohen *d* effect size of 0.89 indicated a large effect. At month 5, engaged participants had a mean weight loss of 11.53% (95% CI 11.5% to 11.6%) compared with 8% (95% CI 7.9% to 8%) in the nonengaged participants (*P*<.001). A Cohen *d* effect size of 0.56 indicated a moderate effect. Participants using tirzepatide achieved more significant weight loss than those using semaglutide at month 5 (13.9%, 95% CI 13.5% to 14.3% vs 9.5%, 95% CI 9.2% to 9.7%; *P*<.001). The proportion of engaged participants achieving ≥5%, ≥10%, and ≥15% weight loss was significantly higher than the nonengaged group at corresponding time points from months 3 to 5 respectively (*P*<.001).

**Conclusions:**

Engagement with a digital weight management platform significantly enhances weight loss outcomes among individuals using GLP-1 receptor agonists. The combination of pharmacotherapy and digital behavioral support offers a promising strategy to promote the supported self-care journey of individuals seeking clinically effective obesity management interventions.

## Introduction

### Background

The global obesity epidemic continues to pose a significant challenge to public health systems worldwide [1]. Obesity is characterized by excessive fat accumulation that impairs health and is associated with an increased risk of multiple noncommunicable diseases, including type 2 diabetes (T2D), cardiovascular diseases, and certain cancers [2]. Beyond individual health consequences, this “disease of the lifestyle” [3] imposes substantial societal and economic burdens due to elevated health care costs and reduced productivity, with a global economic impact estimated at US $1.68 trillion [4].

Traditional management strategies for obesity predominantly focus on lifestyle modifications such as dietary changes, increased physical activity, and behavioral interventions [1,5]. While these approaches are fundamental to initiating weight loss and improving health, they often fail to produce long-term results for many individuals [6]. This limitation is partly due to complex physiological adaptations that occur in response to weight loss, including metabolic slowdown and increased appetite, which traditional interventions may not adequately address [7].

Recent advancements include the introduction of pharmacological interventions to promote obesity management. Glucagon-like peptide-1 (GLP-1) receptor agonists (eg, semaglutide, marketed as Wegovy and Ozempic) [8], and dual GLP-1/gastric inhibitory polypeptide (GIP) receptor agonists like tirzepatide (Mounjaro) [9], represent breakthroughs by targeting physiological pathways that regulate appetite and energy balance. These pharmacotherapies demonstrated the ability to achieve and maintain significant weight loss, ranging from 10% to 20% over 2 years, serving as promising adjuncts to lifestyle interventions [10,11].

Notably, the National Institute for Health and Care Excellence recognized the significant role of digital tools in supporting weight management and provided guidance on the integration of digital health interventions that offer evidence-based behavioral support to individuals aiming to manage their weight effectively [12]. These tools should be designed to promote sustained weight loss through ongoing engagement, personalized feedback, and tailored advice, complementing pharmacological treatments like GLP-1 receptor agonists. The incorporation of such digital platforms aligns with the National Institute for Health and Care Excellence’s broader strategy to enhance the accessibility and effectiveness of obesity management programs across diverse populations while supporting the weight loss journey of individual self-carers who are overweight but otherwise healthy, as well as patients who need to lose weight to tackle multiformity.

However, significant weight loss is not solely a product of medication use but also depends on sustained behavior change and self-care approaches to promote adherence to treatment protocols. Behavioral factors critically influence the success of weight loss interventions [13]. Supported self-management weight loss programs involving pharmacotherapy, such as the digitally enabled Voy, incorporate behavioral change components and use technology to promote end user engagement. By integrating behavior change theories like social cognitive theory [14] and facilitating self-monitoring, goal setting, and feedback mechanisms, these platforms encourage patients to actively manage their health and well-being journey in the community and other settings [15]. Behavioral activation complements weight loss interventions by helping individuals identify and engage in positive, goal-oriented activities that align with their health objectives. It emphasizes breaking the cycle of avoidance or inactivity often associated with obesity, replacing these behaviors with structured, rewarding actions like regular physical activity, healthy eating, and consistent self-monitoring [16]. By focusing on small, achievable steps, behavioral activation builds motivation and self-efficacy, which are crucial for sustained adherence to treatment protocols. When combined with digital tools, such as goal-setting features and real-time feedback, this approach empowers individuals to take ownership of their weight management journey, enhancing both engagement and long-term outcomes [17]. Multicomponent interventions are attractive because they address both the physiological and behavioral aspects of obesity. This approach potentially enhances the effectiveness of pharmacological treatments through ongoing support and education using real-time microlearning approaches to promote individual self-care capability [18], as well as through supporting the adoption of health-seeking self-care behaviors using nudges and personalized insights delivered using an accessible digital platform [19,20].

Clinically significant weight loss is typically defined as a reduction of at least 5% of initial body weight [21], which is associated with improvements in obesity-related comorbidities such as hypertension, dyslipidemia, and insulin resistance [22]. Achieving weight loss of 10% or more is linked to additional health benefits, including enhanced glycemic control, decreased need for diabetic medications, and reduced risk of cardiovascular events [23]. Weight loss of 15% or greater can lead to substantial clinical improvements, such as remission of T2D and significant reductions in cardiovascular risk factors [24]; ultimately interventions in obesity management that aim to reduce weight should ideally also reach thresholds that confer meaningful health benefits.

Digitally supported self-care approaches [25] and supported self-management are integral to lifestyle medicine [26]. Despite the rapid adoption of digital health technologies, there remains a substantial gap in understanding how these digital interventions can complement pharmacological treatments in obesity for improved clinical outcomes compared with pharmacological treatment alone. Randomized controlled trials have examined eHealth platforms with behavioral change coaching for weight loss [27]. However, existing literature is limited, as the integration of digital platforms with pharmacological therapy for weight loss has yet to be extensively researched due to its novelty [28-30].

### Objectives

The aim of this study was to address this gap by exploring the impact of engagement with app-based digital weight loss programs that combine pharmacotherapy using GLP-1 receptor agonists semaglutide and the dual GLP-1/GIP receptor agonist tirzepatide. Considering that the ideal focus is on achieving and sustaining clinically significant weight loss through digital engagement support over specified durations, this study sought to provide valuable insights into the potential and limitations of digital health strategies in enhancing the efficacy of obesity management.

## Methods

### Study Design and Setting

This retrospective 2-arm study was conducted to assess the effectiveness of GLP-1 receptor agonists. Specifically semaglutide (marketed as Wegovy and Ozempic) and the dual GLP-1/GIP receptor agonist tirzepatide (marketed as Mounjaro) within a digitally delivered weight management program. The evaluation used data extracted from the Voy digital health platforms, which were developed to provide remote behavioral support through live group video coaching sessions, text-based in-app support, dynamic educational content, and the direct supply of pharmacotherapy for weight management. The Voy digital health platform was initially created by a multidisciplinary team of clinicians, behavioral scientists, and software developers seeking to create a scalable solution for obesity management. Drawing upon established frameworks in behavior change and self-management support, the platform combined core digital tools (eg, real-time weight monitoring, medication adherence tracking, and personalized coaching sessions) into a single interface accessible via smartphone or web browser.

The study period spanned February 2023 to August 2024, including up to 5 months of data for participants using both semaglutide and tirzepatide. The selection of a 5-month follow-up duration was based on data availability, representing a cross-sectional cohort of individuals who were recently onboarded to the platform for the weight loss service, allowing for a consistent assessment of early treatment outcomes across all participants in the initial 5 months where significant weight loss is typically observed [9]. Furthermore, 5 months was the latest follow-up available for tirzepatide data as the medication service was rolled out in late February 2024.

### Procedure

**Figure 1:**
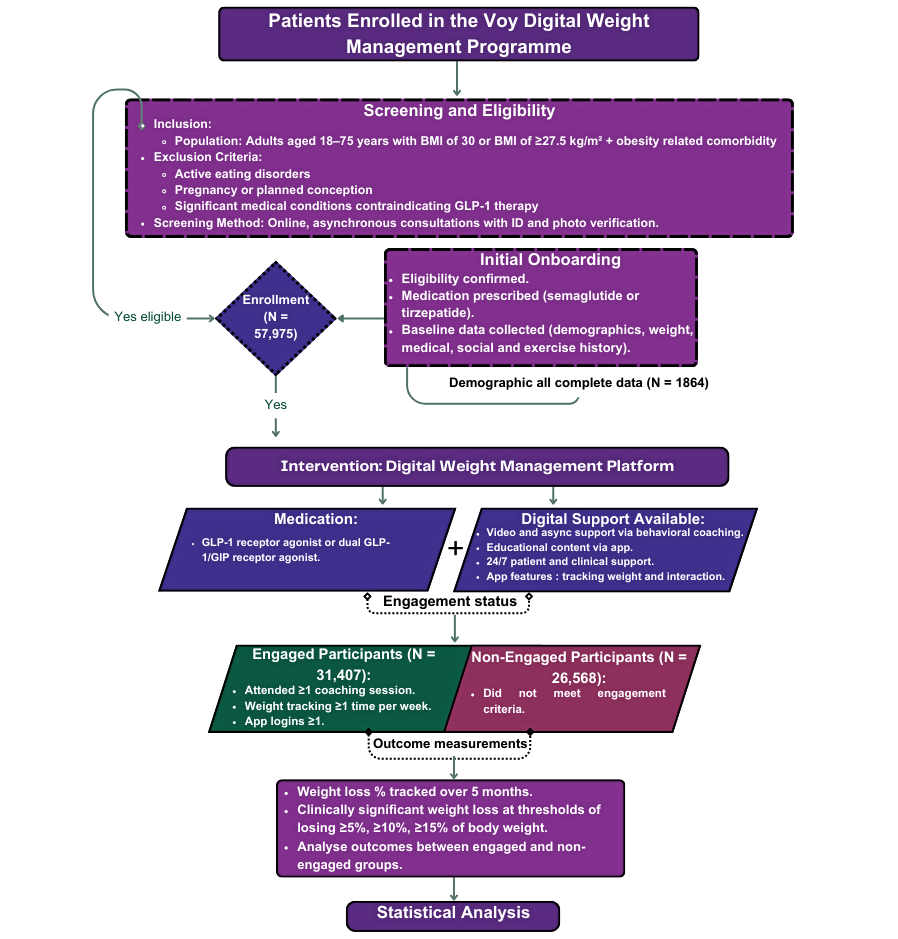
Flowchart illustrating the critical steps of the retrospective analysis conducted for the Voy digital weight management program. GIP: gastric inhibitory polypeptide; GLP-1: glucagon-like peptide-1.

Participants became aware of the Voy program through multiple channels: targeted social media campaigns, physician referrals, word-of-mouth recommendations, and general web searches. They self-enrolled via the Voy website, where they completed a web-based screening questionnaire covering medical history, BMI, and lifestyle factors. Thereafter, the participants interacted with the Voy digital health platform’s weight management program, which integrates GLP-1 receptor and GLP-1/GIP receptor agonist pharmacotherapies with digital behavioral support to enhance weight loss outcomes (Figure 1). Upon enrollment, participants underwent an initial assessment to confirm eligibility, including verification of age, BMI, and absence of exclusion criteria, as outlined in the eligibility criteria below. Participants also completed web-based asynchronous consultations, which included medical suitability checks, identity verification through photo ID, and submission of any required documentation. Baseline demographic information, medical history, and lifestyle factors were requested at the initiation of the program.

Eligible participants received prescriptions for either semaglutide or tirzepatide based on clinical considerations. Medications were delivered directly to participants, and comprehensive medication management was provided through the platform. Instructional materials ensured correct medication administration, and participants had unlimited consultations with guidance and support from a team of clinicians and coaches. The Voy program accessible through the web or via the Voy app, was offered on a paid monthly subscription basis, covering medication, coaching (unlimited call check-ins through texts, audio or video), and supporting resources. The initial monthly cost of enrollment for either semaglutide or tirzepatide was £129 (US $166.70). Prices covered 1 subcutaneous injection pen every 4 weeks, personalized coaching, and access to a web portal with self-tracking tools.

Participants attended group onboarding sessions and were offered fortnightly coaching to enhance engagement and adherence. Coaches were trained based on principles from social cognitive theory, self-determination theory, the transtheoretical model, and the theory of planned behavior [14,31-33]. These techniques focused on fostering intrinsic motivation, goal setting, and problem-solving to promote sustainable lifestyle changes tailored to participants’ individual progress and challenges [20]. Participants were encouraged to actively engage with the app’s dynamic educational content, which was adapted based on their engagement with specific topics, including nutrition, physical activity, and lifestyle factors impacting weight management. The educational content aimed to enhance self-efficacy and equip participants with practical skills to sustain weight loss over time [34].

### Participants

Participants were adult residents of the United Kingdom aged between 18 and 75 years with a BMI of 30 kg/m^2^ or higher or >27.5 kg/m^2^, with the presence of obesity-related comorbidities. All participants initiated a weight management program involving either semaglutide or tirzepatide.

### Eligibility Criteria

Eligibility requires access to a smartphone or tablet to engage with the digital health platform. Exclusion criteria included a history of self-reported eating disorders (eg, anorexia nervosa and bulimia nervosa), pregnancy or active attempts to conceive, known allergies or hypersensitivity to any components of the prescribed medications, and severe medical conditions such as a personal or family history of medullary thyroid carcinoma, multiple endocrine neoplasia syndrome type 2, significant hepatic impairment, renal impairment requiring dialysis, uncontrolled cardiovascular diseases, or severe gastrointestinal disorders (eg, gastroparesis and pancreatitis). Participants who met the eligibility criteria were identified through the digital health platforms’ databases. All eligible individuals who initiated the program within the study period were included, resulting in a sample reflective of real-world clinical practice.

### Data Availability

Engagement metrics and weight loss outcomes were available for the entire sample of 57,975 participants who used the digital platform during the study period. Complete demographic information (eg, age, sex, and ethnicity) was available for a subset of 1864 participants who enrolled during the initial phase of the service. This subset represents the early adopters for whom detailed baseline data were systematically collected to inform program development and initial evaluations.

### Defining Engagement and Outcome

#### Primary Outcome

The primary outcome of this study was the degree of weight loss, measured as the percentage change from each participant’s baseline weight over time. Weight measurements were entered in the app by the participants themselves (ie, self-reported), which we triangulated where possible with additional documentation such as progress photographs.

#### Engagement as an Exposure Variable

##### Overview

Engagement with the digital platform’s program was treated as the primary exposure variable. This engagement was designed to capture the extent of interaction with the platform’s core features, hypothesized to be instrumental in achieving and sustaining weight loss. We operationalized engagement based on 3 key behaviors that the clinical and research teams identified as most likely to drive meaningful outcomes.

##### Coaching Session Attendance

Participation in group or individual coaching sessions (video, audio, or text-based) provided live opportunities for goal-setting, personalized feedback, and motivational support.

##### Weight-Tracking Frequency

Logging weight in the app on a regular basis was encouraged to promote self-monitoring, an evidence-based behavior change strategy shown to correlate with improved weight management outcomes.

##### App Use and Logins

Simply logging into the platform to view educational content, track health metrics, or interact with coaches was considered an important signal of ongoing engagement.

#### Binary Classification for Analysis

##### Overview

Although these 3 components can each vary in intensity (eg, how many coaching sessions, how frequently weight was logged, app login outside weight logging), for the primary analysis we simplified the categorization into “engaged” or “nonengaged,” as determined in consultation with the clinical team:

##### Engaged

Met any one (or more) of the following three criteria at least once during the study period: (1) attended ≥1 weight coaching session (group or individual), (2) tracked weight ≥1 per week in the app, or (3) logged into the app (for features other than only weight logging) at least once for the duration of the program.

##### Nonengaged

Participants who did not fulfill any of the above criteria (note: although nonengaged participants did not perform regular weight logging, a minimal weight tracking value [eg, at least one per month] was still required for inclusion in the aggregate mean percentage change calculation).

We selected “≥1 coaching session” to detect minimal real-time professional support or group interaction, weekly weigh-ins to align with standard clinical recommendations without becoming overly burdensome, and at least one app login to capture a basic level of platform use, thereby establishing a minimal yet meaningful threshold to differentiate participants with any engagement from those entirely disengaged.

### Variables

The primary outcome of the study was the percentage weight change from baseline, calculated as the aggregated average of each individual’s weekly weight change converted to a monthly average (if recorded at least once a month) and then pooled across all participants. This outcome included the effect of weight loss medication use, reaching clinically significant weight loss thresholds defined as loss of ≥5%, ≥10%, and ≥15% of baseline weight. Engagement with the digital platform was assessed as an exposure variable. Other exposure variables included the type of medication used, categorized as semaglutide or tirzepatide.

### Statistical Analysis

Descriptive statistics were used to summarize baseline characteristics, with means and SDs for continuous variables and frequencies and percentages for categorical variables. Chi-square tests were used to compare the proportion of participants achieving clinically significant weight loss (≥5%, ≥10%, and ≥15% of baseline weight) between the engaged and nonengaged groups. In cases where small sample sizes or low expected frequencies were present (ie, n<5), Fisher exact test was used. Odds ratios (ORs) and 95% CIs were calculated to quantify the strength of the association between engagement and weight loss success through 2×2 contingency tables.

To assess the time-to-event data for achieving clinically significant weight loss, Kaplan-Meier survival analysis was performed, comparing the time to reach ≥5%, ≥10%, and ≥15% weight loss between the engaged and nonengaged groups. The log-rank test was used to evaluate the statistical significance of differences between the survival curves of the 2 groups. This approach allowed us to visualize the cumulative incidence of weight loss over time and assess the role of engagement in accelerating weight loss success.

An independent sample *t* test was used to compare the mean weight change between the engaged and nonengaged groups. Cohen *d* was calculated to determine the effect size, providing insight into the magnitude of differences between groups. Prior to conducting the *t* test, assumptions of normality and homogeneity of variances were assessed using the Shapiro-Wilk test. When assumptions were violated, a nonparametric alternative, the Mann-Whitney U test, was considered.

All statistical analyses were performed using R (version 4.3.1; R Foundation for Statistical Computing) and its statistical packages. A significance level of *P*<.05 was set for all statistical tests.

### Sample Size Calculation

A sample size calculation was performed to estimate the number of participants required to detect a significant difference in weight loss between engaged and nonengaged groups. Using an estimated effect size of a 15% difference in the proportion of participants achieving ≥10% weight loss between groups, with an expected 30% of engaged participants and 15% of nonengaged participants achieving this outcome, a minimum of 118 participants per group was calculated to provide 80% power at a 5% significance level. To account for attrition, a target sample size of 250 participants per group was set.

Baseline demographic information, medical history, and lifestyle factors were requested at the initiation of the program. Data concerning medication adherence were monitored through self-reports and prompted reminders. All data were anonymized upon extraction to ensure confidentiality and compliance with the General Data Protection Regulation.

### Sensitivity Analysis

In addition to the primary unadjusted analyses, a sensitivity analysis was conducted on the subset of participants with complete baseline data (N=1864). We examined the mean percentage weight change by engagement status. To account for potential confounders, we conducted a multivariable linear regression for each monthly time point (months 1 to 5). To further assess the robustness of our findings, we conducted a multivariable-adjusted sensitivity analysis. Specifically, we examined the association between engagement status (engaged vs nonngaged) and weight loss at each month (months 1 to 5) using ordinary least squares regression, controlling for potential confounders We regressed percentage weight change (%) from baseline on engagement status (engaged vs nonengaged), adjusting for clinical indicators such as age, gender, baseline weight, BMI category, presence of T2D, presence of high cholesterol, and presence of high blood pressure. For each monthly time point, the outcome variable was the percentage weight change from baseline. The corresponding results are presented in Tables S1 and S2 in Multimedia Appendix 1.

### Bias and Missing Data

Self-reported weight measurements could introduce reporting bias. To mitigate this, participants were encouraged to provide accurate measurements through regular reminders and had the option to upload progress photographs, enhancing data validity. Additionally, data validation checks were performed to identify and address implausible values. Selection bias was minimized by including all eligible participants who initiated the program within the study period, ensuring the sample was representative of the population using these services.

### Ethical Considerations

This study (ICREC#7363051) was reviewed by the Imperial College Research Ethics Committee, which granted a favorable opinion, signifying that the Committee determined the study to be ethically acceptable and in alignment with institutional and regulatory guidelines. As this study used secondary analysis of anonymized data collected during routine care, formal ethical approval from the National Health Service Research Ethics Committee was not required under National Health Service standards for service evaluations. The study adhered to the principles outlined in the Declaration of Helsinki. Participants provided informed consent for their anonymized data to be used for research and service improvement purposes upon enrollment in the program, the ICREC has approved secondary analysis of anonymized data without additional consent. Data protection and confidentiality were strictly maintained in compliance with General Data Protection Regulation regulations.

### Adherence to Guidelines

To improve the quality of the reporting, this study adhered to the STROBE (Strengthening the Reporting of Observational Studies in Epidemiology) guidelines [35].

## Results

### Participant Characteristics

A total of 57,975 participants were included in the engagement and outcome analyses. Among these, 31,407 (54.2%) participants were classified as engaged and 26,568 (45.8%) participants as nonengaged at baseline (month 0). Due to changes in data collection practices over time and staggered onboarding during the study period, the number of participants reporting weight data decreased at subsequent time points, comprehensive demographic and clinical characteristics were available for a subset of 1864 participants who enrolled at the inception of the service. The characteristics of this subset are presented in Table 1.

**Table 1:** Displays demographic, clinical, and lifestyle characteristics of participants with complete baseline data for the service. The “overall” group includes all 1864 participants, while the “engaged” versus “nonengaged” columns show how these characteristics differ by engagement status at baseline.

Characteristic				Overall (n=1864)			Engaged (n=1520)		Nonengaged (n=344)
Age (years), mean (SD)				45.2 (10.5)			45.8 (11.6)		44.4 (11.9)
**Sex, n (%)**									
	Female		1598 (85.7)			1316 (82.4)			282 (17.6)
	Male		266 (14.3)			204 (76.7)			62 (23.3)
BMI (kg/m²), mean (SD)				36.0 (6.3)			36.1 (6.4)		35.6(5.9)
Weight (kg), mean (SD)				100.9 (6.3)			101.3 (20.4)		99.5 (19.4)
**Ethnicity, n (%)**									
	White British/Irish		1442 (77.4)			1194 (82.8)			248 (17.2)
	White other		199 (10.7)			150 (75.4)			49 (25)
	Asian Indian		39 (2)			30 (77)			9 (23)
	Asian Pakistani		21 (1)			19 (91)			2 (10)
	Black African		31 (2)			21 (68)			10 (32)
	Other ethnic groups		132 (7.1)			106 (80.3)			26 (20)
**Comorbidities, n (%)**									
	Type 2 diabetes		79 (4)			64 (4.2)			15 (4)
	High blood pressure		289 (15.5)			239 (15.7)			50 (15)
	High cholesterol		192 (10.3)			163 (10.7)			29 (8)
	Osteoarthritis		134 (7.2)			117 (7.7)			17 (5)
	Chronic back pain		165 (8.9)			146 (9.6)			19 (6)
	Depression		352 (18.9)			293 (19.3)			59 (17)
**Lifestyle factors, n (%)**									
	**Smoking status**								
		Never smoked	946 (50.8)		768 (50.5)			178 (51.7)	
		Ex-smoker	584 (31.3)		484 (31.8)			100 (29.1)	
		Current smoker	102 (5.5)		79 (5)			23 (7)	
		Not answered	232 (12.4)		189 (12.4)			43 (13)	
	**Alcohol consumption/weekly**								
		None (0 units)	766 (41.1)		622 (40.9)			144 (41.9)	
		1-6 units	656 (35.2)		543 (35.7)			113 (32.8)	
		≥7 units	442 (23.7)		355 (23.4)			87 (25)	
	**Dietary habits**								
		Balanced diet	1271 (68.2)		1050 (69.1)			221 (64.2)	
		High in animal products	335 (18.0)		268 (17.6)			67 (20)	
		Vegetarian/vegan	258 (13.8)		202 (13.3)			56 (16)	
	**Physical activity**								
		Daily	180 (9.7)		152 (10.0)			28 (8)	
		Regularly (3-4 times/week)	623 (33.4)		514 (33.8)			109 (31.7)	
		Occasionally (1-2 times/week)	703 (37.7)		568 (37.4)			135 (39.2)	
		Rarely/never	358 (19.2)		286 (18.8)			72 (21)	
		Family history of obesity	991 (53.2)		812 (53.4)			179 (52.1)	
	**Medications, n (%)**								
		Blood pressure	289 (15.5)		239 (15.7)			50 (14.5)	
		Cholesterol	192 (10.3)		163 (10.7)			29 (8)	
		Diabetes	79 (4)		64 (4)			15 (4)	
		Thyroid	135 (7.2)		114 (6.1)			21 (1)	
		Antidepressants	469 (25.2)		388 (25.6)			81 (24)	

### Engagement Levels

At baseline (month 0), among the 57,975 participants who tracked their weight, 31,407 (54.2%) were engaged, and 26,568 (45.8%) were nonengaged. Engagement levels decreased over time, with 3622 (6.3%) engaged participants remaining at month 5 (Table 2).

**Table 2:** Mean percentage of weight loss by engagement in participants taking medication and using the digital platform. All weight loss values are mean aggregates and are in a negative value.

Month from medication start and engagement		Mean percentage weight lost (95% CI)	Absolute percentage point difference		*P* value^a^		Effect size (*d*)		Relative percentage difference		Number of patients		Total patients	
**0**				0.00		N/A^b^		0.00		N/A				57,975
	No	N/A									26,568			
	Yes	N/A									31,407			
**1**				0.97		<.001		0.179		33.8				23,306
	No	2.87 (2.86-2.88)									1429			
	Yes	3.84 (3.83-3.85)									21,877			
**2**				1.88		<.001		0.291		37.4				14,826
	No	5.03 (5.01-5.05)									1848			
	Yes	6.90 (6.89-6.91)									12,978			
**3**				3.11		<.001		0.891		52.4				10,120
	No	5.93 (5.90-5.96)									1691			
	Yes	9.04 (9.02-9.06)									8429			
**4**				3.68		<.001		0.616		52.7				6826
	No	6.99 (6.95-7.03)									1331			
	Yes	10.67 (10.64-10.70)									5495			
**5**				3.53		<.001		0.560		44.3				4999
	No	7.97 (7.93-8.01)									1377			
	Yes	11.50 (11.49-11.57)									3622			

^a^*P* values derived from independent *t* test.

^b^Not applicable.

### Weight Loss Outcomes

#### Weight Loss by Engagement Status

**Figure 2:**
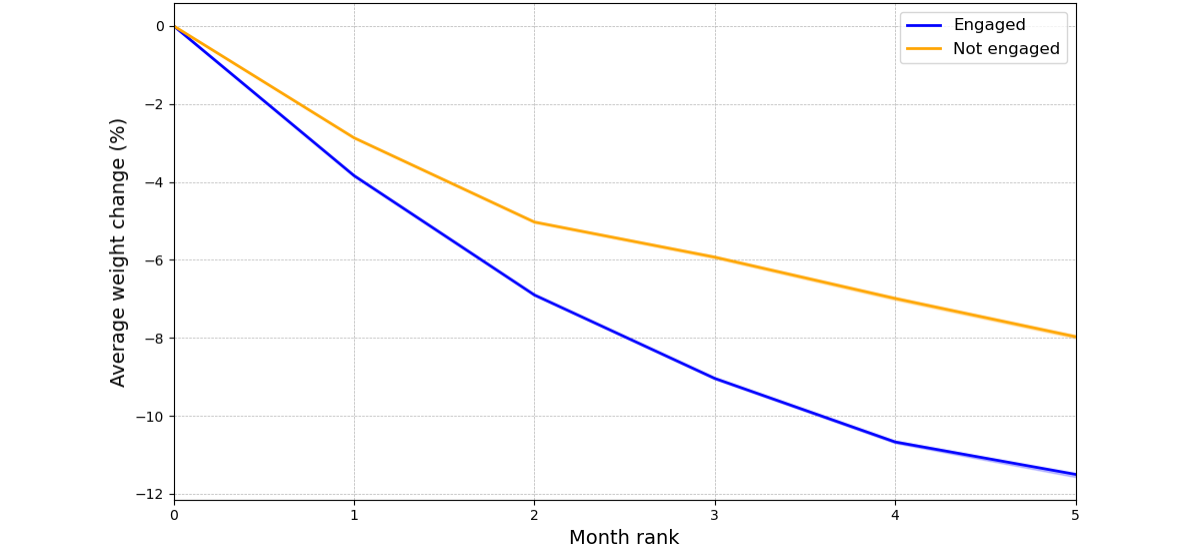
Average weight loss (kg) trajectory over time by engagement status.

Engaged participants consistently achieved greater weight loss compared with nonengaged participants at each time point (Figure 2). At month 1, engaged participants experienced a mean weight loss of 3.8% (95% CI 3.9% to 3.8%), whereas nonengaged participants had a mean weight loss of 2.9% (95% CI 2.9% to –2.9%), a significant difference (*P*<.001). This trend persisted over subsequent months. By month 5, engaged participants had a mean weight loss of 11.5% (95% CI 11.6% to 11.5%), while nonengaged participants had a mean weight loss of 8% (95% CI 8% to 7.9%), a significant difference of 3.56 percentage points (*P*<.001).

#### Subgroup Sensitivity Analysis

A subgroup of 1864 participants with available demographic and baseline data allowed for a more detailed sensitivity analysis (Table S1 in Multimedia Appendix 1). Overall, engaged participants consistently had a significantly greater mean percentage weight loss than nonengaged participants from month 1 to month 5 (all *P*<.05). In a separate multivariable-adjusted regression model (Table S2 in Multimedia Appendix 1), engagement remained a strong predictor of increased weight loss across all 5 months (*P*<.001), even after adjusting for age, gender, baseline weight, BMI category, and obesity-related comorbidities. Higher baseline BMI (overweight or obese) also showed a notable association, whereas the effects of T2D and other comorbidities varied by month.

#### Weight Loss by Medication Type

**Figure 3:**
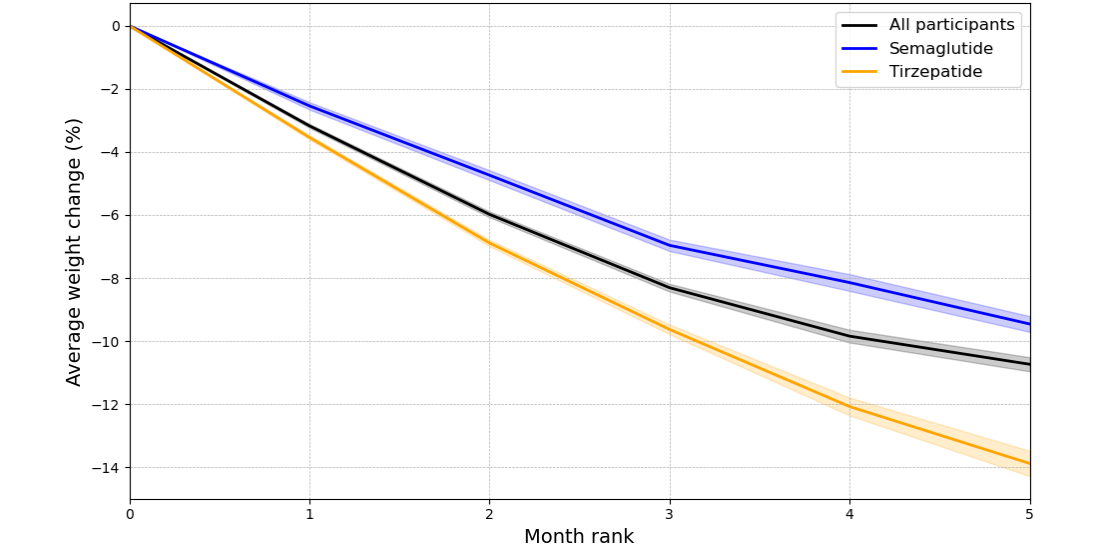
Depicts the mean percentage of weight loss over time by medication type. “All participants” shows the combined weight loss trajectory for both tirzepatide and semaglutide users.

Participants were prescribed either semaglutide or tirzepatide (Mounjaro). Weight loss trajectories differed significantly between the 2 medication groups over the study period (Figure 3, Table S3 in Multimedia Appendix 2). At month 5, participants using tirzepatide achieved a mean weight loss of 13.9% (95% CI 14.3% to 13.5%), significantly greater than the 9.5% (95% CI 9.7% to 9.2%) observed in semaglutide users (*P*<.001).

#### Proportion Achieving Clinically Significant Weight Loss

The proportion of participants achieving clinically significant weight loss thresholds (≥5%, ≥10%, and ≥15% of baseline weight) was higher among engaged participants compared with nonengaged participants. At the ≥5% weight loss threshold, engaged participants had significantly higher odds of achieving this goal compared with nonengaged participants at all assessed time points. Table 3 presents the detailed ORs for achieving ≥5% weight loss over time.

For the ≥10% weight loss threshold, significant differences emerged from month 2 onward. Table 4 provides the ORs for achieving ≥10% weight loss over time.

At the ≥15% weight loss threshold, significant differences were observed at months 3 and 4. Table 5 details the ORs for achieving ≥15% weight loss over time.

**Table 3:** Odds ratios (ORs) for achieving ≥5% weight loss by engagement status.

Month	Engaged; achieved goal, n (%)	Engaged; did not achieve goal, n (%)	Nonengaged; achieved goal, n (%)	Nonengaged; did not achieve goal, n (%)	OR (95% CI)	*P* value^a^
1	3083 (15.1)	17,332 (84.9)	104 (8.9)	1066 (91.1)	1.82 (1.49-2.24)	<.001
2	6386 (48.9)	6678 (51.1)	140 (18.6)	612 (81.4)	4.18 (3.47-5.04)	<.001
3	5454 (60.8)	3522 (39.2)	136 (27.9)	352 (72.1)	4.01 (3.27-4.91)	<.001
4	3850 (63.7)	2198 (36.3)	99 (32)	214 (68.4)	3.79 (2.97-4.83)	<.001
5	2664 (61.0)	1705 (39.0)	69 (37)	117 (62.9)	2.65 (1.96-3.59)	<.001

^a^*P* value derived from chi-square test.

**Table 4:** Odds ratios (OR) for achieving ≥10% weight loss by engagement status.

Month	Engaged; achieved goal, n (%)	Engaged; did not achieve goal, n (%)	Nonengaged; achieved goal, n (%)	Nonengaged; did not achieve goal, n (%)	OR (95% CI)	*P* value^a^
1	331 (1.6)	20,084 (98.4)	25 (2)	1145 (97.9)	0.75 (0.50-1.14)	.22
2	1246 (9.5)	11,818 (90.5)	26 (4)	726 (96.5)	2.94 (1.98-4.37)	<.001
3	2378 (26.5)	6598 (73.5)	51 (11)	437 (89.5)	3.09 (2.30-4.14)	<.001
4	2217 (36.7)	3831 (63.3)	39 (13)	274 (87.5)	4.07 (2.90-5.71)	<.001
5	1722 (39.4)	2647 (60.6)	34 (18)	152 (81.7)	2.91 (2.00-4.24)	<.001

^a^*P* value derived from chi-square test.

**Table 5:** Odds ratios (OR) for achieving ≥15% weight loss by engagement status.

Month	Engaged; achieved goal, n (%)	Engaged; did not achieve goal, n (%)	Nonengaged; achieved goal, n (%)	Nonengaged; did not achieve goal, n (%)	OR (95% CI)	*P* value^a^
1	93 (1)	20,322 (99.5)	12 (1)	1158 (99.0)	0.44 (0.24-0.81)	.01
2	176 (1.3)	12,888 (98.7)	11 (1.5)	741 (98.5)	0.92 (0.50-1.70)	.92
3	509 (5.7)	8467 (94.3)	9 (2)	479 (98.2)	3.20 (1.64-6.22)	<.001
4	793 (13.1)	5255 (86.9)	14 (4.6)	299 (95.5)	3.22 (1.88-5.54)	<.001
5	752 (17.2)	3617 (82.8)	20 (11)	166 (89.2)	1.73 (1.08-2.76)	.03

^a^*P* value derived from chi-square test.

#### Time to Achieve Weight Loss Thresholds

**Figure 4:**
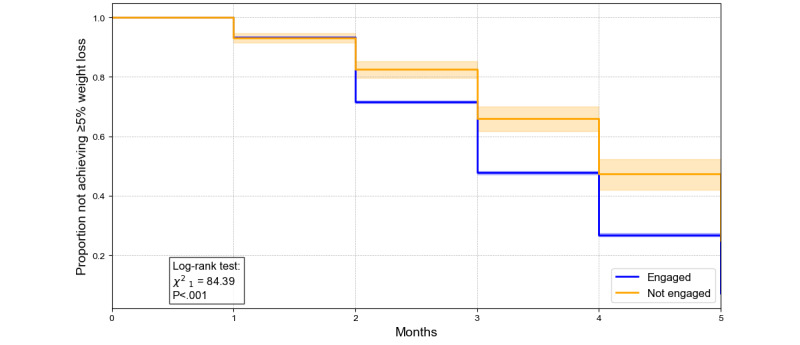
Kaplan-Meier survival curves for time to achieve ≥5% weight loss by engagement status. The y‐axis displays the proportion of participants who have not yet reached ≥5% weight loss since baseline. The x‐axis shows time in months from program initiation (ie, when participants began pharmacotherapy and were enrolled in the digital program). The orange line (with shading representing the 95% CI) represents the engaged group, and the blue line (with 95% CI shading) represents the nonengaged group. A log-rank test (χ^2^_1_=38.39; *P*<.001) compared the curves over 5 months, revealing a statistically significant difference in the rate at which each group achieved ≥5% weight loss.

**Figure 5:**
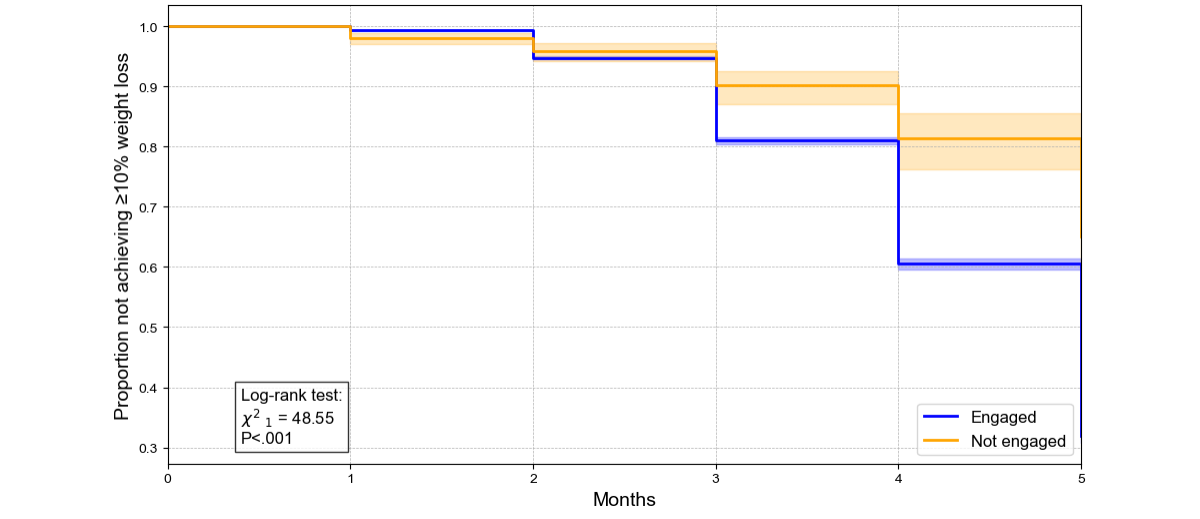
Kaplan-Meier survival curves for time to achieve ≥10% weight loss by engagement status. The y-axis is the proportion of participants who have not reached ≥10% weight loss, and the x-axis is the time (months) from the program start. The orange line/shading denotes the engaged group; the blue line/shading denotes the nonengaged group. The log-rank test (χ^2^_1_=45.65; *P*<.001) indicates a significant difference in time to ≥10% weight loss across the 2 engagement groups.

**Figure 6:**
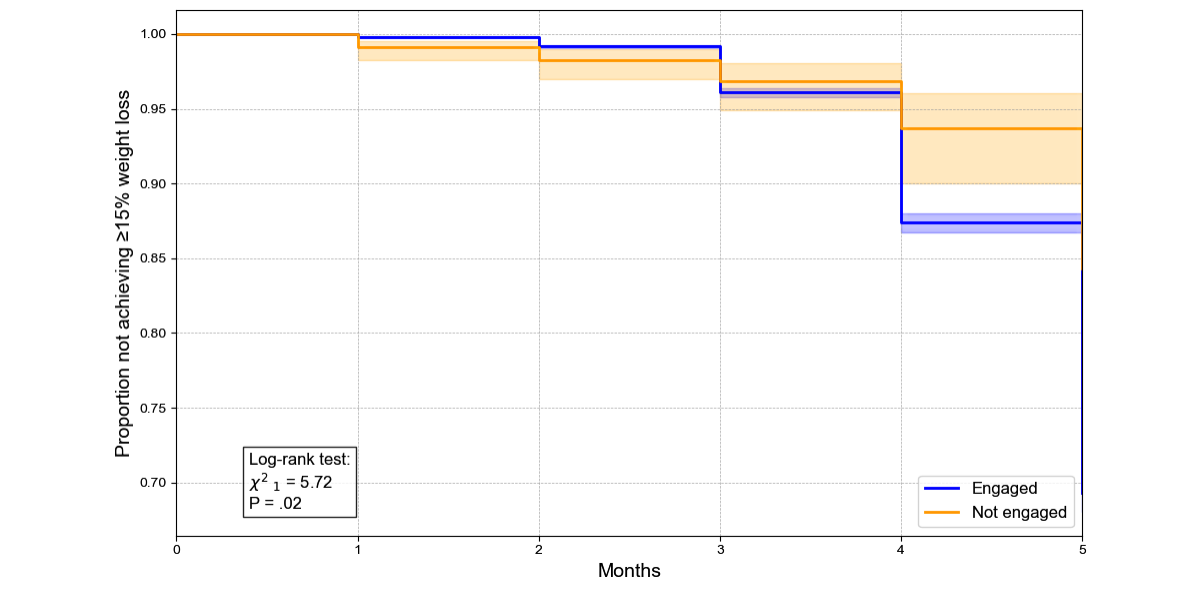
Illustrates the Kaplan-Meier survival curves for time to achieve ≥15% weight loss by engagement status. The y-axis shows the proportion of those who have not achieved ≥15% weight reduction, and the x-axis shows the number of months of follow-up. The orange line/shading denotes the engaged group; the blue line/shading denotes the nonengaged group. The log-rank test (χ^2^_1_ = 17.72; *P*<.001) confirms a significant difference in time to ≥15% weight loss between the 2 groups.

Kaplan-Meier analyses (Figures 4-6) revealed significant differences in the proportions of individuals achieving 5%, 10%, and 15% weight loss over 5 months between engaged and nonengaged participants. For example, at month 4, the proportion not achieving the 5% threshold was 49.3% for engaged participants compared with 69.8% for nonengaged participants (*P*<.001). Similarly, by month 5, 59.9% of engaged participants had yet to achieve the 10% threshold compared with 81.5% of nonengaged participants (*P*<.001). For the 15% threshold, while differences were less pronounced, 86.5% of engaged participants had not reached this target by month 5, compared with 93.5% of nonengaged participants (*P*=.02). These findings underscore the role of engagement in facilitating weight loss, with the effect diminishing at higher thresholds.

## Discussion

### Principal Results

This study provides compelling evidence that engagement with digital health platforms significantly enhances weight loss outcomes among individuals undergoing pharmacotherapy with GLP-1 receptor agonists for obesity management. Engaged participants achieved greater mean weight loss over time and reached clinically significant weight loss thresholds more rapidly than their nonengaged counterparts. The superior efficacy of tirzepatide over semaglutide observed in this real-world study aligns with findings from recent clinical trials reinforcing the potential of dual agonist therapies in obesity management [36].

### Limitations

The principal limitation of this study was the potential selection bias, as participants who chose to engage with the digital platform may inherently possess higher motivation levels or greater health literacy. This could confound the relationship between engagement and weight loss outcomes. Although we sought to minimize this selection bias by including all eligible participants, unmeasured confounding must be considered. The discrepancy in available demographic data and the reduction in participant numbers over time may also introduce attrition bias. We also acknowledge that missing data on key variables limits the ability to adjust for confounders and affects the representativeness of the sample.

Although we used a binary engaged version nonengaged framework for the main analysis, we recognize that engagement can be multifaceted. Future or secondary analyses might subdivide participants into multiple tiers, such as low, moderate, or high engagement based on the frequency of each component (eg, “attended ≥5 coaching sessions” or “logged weight ≥3 times per week”). This would allow a “dose-response” examination of how incremental increases in engagement correspond to weight loss outcomes.

The discrepancy in demographic data collection reflects adaptations made during program scaling to improve accessibility. Initially, comprehensive data were collected; however, this was streamlined to simplify onboarding and enhance user experience, prioritizing essential data for engagement analysis. While this approach limits detailed subgroup analysis, the broader data set ensures robust insights into program effectiveness. The collection of data and availability of participant information may account for reducing participant numbers at time intervals. Also, participants may have chosen to no longer engage with the provider. This was retrospective data, and missing observations are more common [37].

Reliance on self-reported weight data also introduces the potential for reporting bias, as participants may underreport or overreport their weight due to social desirability or recall bias. The provision of options to upload progress photographs may mitigate this but does not eliminate the issue. Crucially, the absence of a randomized control group limits the ability to establish causality definitively. Observed associations may also be influenced by unmeasured confounding variables or external factors. Pertinently, the maximum follow-up period of 5 months may not capture long-term weight maintenance or the sustainability of engagement with the digital platform. Given that weight regain is a common challenge in obesity management, longer-term studies are needed to assess enduring effects. Therefore, future studies with extended follow-up durations are warranted to clarify whether these benefits endure and to evaluate ongoing engagement.

The study population was predominantly female and of White British/Irish ethnicity, which also limits the generalizability of the findings to more diverse populations as cultural, socioeconomic, and gender differences may influence engagement and weight loss outcomes. Thus, the impact of restricted access to technology and potential barriers to preventing engagement from a health equity perspective must be considered in future studies and policymaking. Additionally, whereas only a subset of 1864 participants was available for analysis, we acknowledge that this subset may not likely be representative of the total population.

We also acknowledge that the requirement for smartphone or tablet access and the monthly subscription fee (the services require payment) may exclude individuals from lower socioeconomic backgrounds or older adults less comfortable with technology, potentially exacerbating health disparities.

Building on the insights from this study, future research should conduct randomized controlled trials comparing pharmacotherapy with and without digital behavioral support to establish causality and quantify the added benefit of digital engagement. Qualitative studies investigating user experiences, barriers to engagement, and facilitators of sustained use can inform the design of more effective digital interventions. Longitudinal studies with extended follow-up periods are needed to evaluate the sustainability of weight loss and the impact of ongoing engagement on weight maintenance and metabolic health. Efforts to collect comprehensive demographic and clinical data for all participants will improve the ability to adjust for confounding factors and understand the differential effects across subgroups.

Research should focus on strategies to streamline timely access to supported self-management technologies, including pharmacotherapy, cognitive behavior therapy approaches, and digital self-care interventions among diverse populations, including those with limited digital literacy, to ensure equitable benefits and reduce health disparities. Incorporating microdata from wearables, biomarker assessments, and validated scales for psychological measures, quality of life, and individual self-care capability can enhance the accuracy of outcomes and provide a more holistic evaluation of health improvements.

### Comparison With Prior Work

The integration of supported self-care interventions involving pharmacotherapy into obesity management represents a major shift that can help address the various challenges associated with weight loss and maintenance. Digital platforms offer scalable, personalized, and accessible supported self-management solutions that transcend traditional barriers to health care delivery [38]. In this example, the digital platforms facilitated self-monitoring, behavioral coaching, and personalized feedback components that are critical to promoting sustained behavior change. The theoretical foundations of digital interventions are grounded in established behavior change models such as the transtheoretical model and self-determination theory [39], emphasizing self-efficacy, intrinsic motivation, and readiness to change [40]. By providing real-time feedback and reinforcing positive behaviors, digital platforms can strengthen self-regulatory processes essential for weight management [41].

The increasing pervasiveness of mobile health technologies facilitates continuous engagement and data collection [42] and presents an unprecedented opportunity for self-driven health care approaches to deliver interventions at scale and reach populations traditionally underserved or facing barriers to in-person care [25]. The enhanced weight loss observed among engaged participants highlights the potential of digital platforms to augment traditional treatment modalities.

Several synergistic mechanisms likely contributed to the enhanced weight loss observed among engaged participants. Enhanced self-monitoring and accountability are fundamental, as frequent tracking of weight and behaviors is a consistent predictor of weight loss success [43]. Digital self-care platforms simplify this process through user-friendly interfaces and automated reminders, reducing the burden of manual tracking and increasing adherence [36]. Tailored interventions have been shown to be more effective than generic advice in promoting behavior change for weight loss [44]. This is likely because personalized behavioral support provided through coaching and tailored feedback also addresses individual barriers and facilitators to weight loss, further enhancing motivation and self-efficacy [45]. The principles of behavioral activation with a focus on action following analysis of behaviors enables a structured problem-solving approach that provides a treatment foci related to change [46]. This incorporation of evidence-based behavior change techniques, such as goal setting, action planning, and problem-solving, is associated with improved weight loss outcomes [47]. Features enabling interaction with peers or health professionals provide social support, which is a critical determinant of weight management success [48], whereas social comparison and normative influence may further motivate adherence to weight loss plans [49]. Regular engagement with the app may also facilitate the formation of automatic healthy behaviors through repetition and reinforcement, as suggested by habit formation theory [50]. Combining behavioral interventions with pharmacotherapy may produce a synergistic effect, enhancing the biological mechanisms of weight loss medications through improved adherence and lifestyle modifications [51].

Our findings align with and extend existing literature on integrating digital interventions with pharmacological treatments for obesity. Previous studies have demonstrated that digital behavioral interventions can enhance the effectiveness of weight loss medications [52]. Furthermore, as research has suggested the improvement in engagement through wearables, and ultimately health, this facet of digital health should be considered for weight loss, as better engagement through digital tools may improve health outcomes. For example, a meta-analysis by Beleigoli et al [53] found that web-based interventions resulted in modest but significant additional weight loss compared with standard care. Moreover, our findings broaden the current literature on digital weight loss interventions that incorporate pharmacotherapy, building on recent work by Richards et al [29,30] which demonstrated that a remotely delivered, semaglutide-supported weight management program is both effective and efficacious in the short term. Our study, however, underscores the use of tirzepatide in a remote setting, yielding superior weight loss outcomes relative to semaglutide as evidenced in clinical trials [54,55]. Furthermore, Talay et al [56] have shown that proactive, personalized lifestyle coaching design significantly enhances patient engagement in semaglutide-supported programs, while another study by Talay et al [57] highlights the robust efficacy of tirzepatide-supported digital interventions [58]. We acknowledge that the dual action on GIP and GLP-1 receptors may confer additive or synergistic effects on glycemic control and appetite regulation, leading to greater weight reductions [59]. Additionally, while Clark et al [57] reported that 64.2% of participants achieved a clinically significant ≥5% weight loss by 12 months, nearly 70% of our engaged participants reached this milestone by month 5. These findings highlight the enhanced potential of remotely delivered, engagement-driven weight loss platforms to achieve scalable, sustained outcomes in obesity management.

### Conclusions

This study provides compelling evidence that structured engagement with a digital self-management platform significantly enhances weight loss outcomes by as much as 53% at month 4 in engaged individuals undergoing pharmacotherapy for obesity. The integration of digital behavioral support with pharmacological treatments represents a synergistic approach that addresses both the biological and behavioral dimensions of obesity. By facilitating self-monitoring, providing personalized feedback, and using nudge and gamification to promote sustained engagement, digital self-management tools can amplify the effectiveness of pharmacotherapies, thus the use of wearables and enhanced user interaction through analytics-driven feedback systems can aid in better engagement. As the prevalence of obesity continues to rise globally, innovative self-driven health care solutions that leverage technology to support behavior change are needed to scale benefits to individuals with noncommunicable diseases, and so-called diseases of the lifestyle.

## Appendix

Multimedia Appendix 1 Sensitivity analysis of mean percentage weight change by engagement status among users with complete baseline data, and multivariable-adjusted ordinary least squares regression of percentage weight change from baseline among users with complete baseline data.

Multimedia Appendix 2 Detailed summary of weight change data across six time points (Month Rank 0 to 5) for three groups: All participants, those on Semaglutide, and those on Mounjaro.

## Abbreviations

GIP: gastric inhibitory polypeptide
GLP-1: glucagon-like peptide-1
OR: odds ratio
STROBE: Strengthening the Reporting of Observational Studies in Epidemiology
T2D: type 2 diabetes

## Data Availability

The data sets generated or analyzed during this study are not publicly available due to the nature of the clinical data collected and the consent provided by patients. The individual participant data used in this study is not publicly available. The statistical code used in the analyses can be made available to researchers upon request to the corresponding author. Requests for access to anonymized data for noncommercial projects should be directed to the corresponding author and will require appropriate ethical approvals, but are available from the corresponding author on reasonable request.
