# Supplementary material for: Impact of Digital Engagement on Weight Loss Outcomes in Obesity Management Among Individuals Using GLP-1 and Dual GLP-1/GIP Receptor Agonist Therapy: Retrospective Cohort Service Evaluation Study
Source: J Med Internet Res. 2025 Mar 31;27:e69466. doi: 10.2196/69466 (PMC11997532; doi:10.2196/69466)
Supplement: Multimedia Appendix 1 [file jmir_v27i1e69466_app1.docx]

| **Month** | **Overall N** | **Overall Mean Weight Change (%) (95% CI)** | **Engaged N** | **Engaged Mean Weight Change (%) (95% CI)** | **Non-Engaged N** | **Non-Engaged Mean Weight Change (%) (95% CI)** | **P-Value*** | **Cohen’s *d*** |
| --- | --- | --- | --- | --- | --- | --- | --- | --- |
| **0** | 1,864 | 0 | 1,525 | 0 | 339 | 0 | — | — |
| **1** | 1,561 | −4.12 (−4.26 to −3.98) | 1,354 | −4.21 (−4.35 to −4.07) | 207 | −3.54 (−4.03 to −3.04) | 0.0104 | −0.21 |
| **2** | 1,570 | −7.33 (−7.52 to −7.14) | 1,366 | −7.44 (−7.64 to −7.24) | 204 | −6.57 (−7.21 to −5.94) | 0.0106 | −0.21 |
| **3** | 1,754 | −9.91 (−10.13 to −9.69) | 1,442 | −10.22 (−10.45 to −9.99) | 312 | −8.47 (−9.05 to −7.88) | <.001 | −0.36 |
| **4** | 1,421 | −12.73 (−13.00 to −12.46) | 1,241 | −12.95 (−13.24 to −12.67) | 180 | −11.20 (−12.07 to −10.32) | <.001 | −0.32 |
| **5** | 1,327 | −14.99 (−15.30 to −14.68) | 1,165 | −15.24 (−15.57 to −14.92) | 162 | −13.18 (−14.14 to −12.22) | <.001 | −0.35 |

Table S1. Sensitivity analysis of mean percentage weight change by engagement among the subgroup with complete baseline data (n = 1,864). P values* derived from independent *t* tests comparing engaged vs non-engaged at each month. Negative signs (−) indicate weight loss relative to baseline.

Table S2. Multivariable-adjusted ordinary least squares (OLS) regression of percentage weight change from baseline, by month (subgroup n = 1,864 with complete baseline data). Multivariable-adjusted regression of percentage weight change from baseline, by month. OLS coefficients (β) represent the change in mean % weight loss relative to the reference group (for categorical variables) or per 1-unit increase (for continuous variables), holding other covariates constant. Reference categories: female (gender), no diabetes, no high cholesterol, no high blood pressure, normal Body Mass Index (BMI) group. The dependent variable is percentage weight change from baseline at each month. *β** coefficients represent the difference in percentage weight change relative to the reference group (for categorical variables) or per 1-unit increase (for continuous variables), holding other covariates constant.

| **Predictor** | **Month 1 β* (P-Value)** | **Month 2 β* (P-Value)** | **Month 3 β* (P-Value)** | **Month 4 β* (P-Value)** | **Month 5 β* (P-Value)** |
| --- | --- | --- | --- | --- | --- |
| **Intercept** | -8.71 (<.001) | -11.55 (<.001) | -11.23 (<.001) | -13.05 (<.001) | -11.59 (<.001) |
| **Engagement (1=Engaged)** | -0.71 (<.001) | -0.88 (0.001) | -1.60 (<.001) | -1.58 (<.001) | -1.94 (<.001) |
| **Gender (male)** | 0.09 (0.668) | 0.50 (0.071) | 0.69 (0.024) | 1.20 (0.001) | 2.07 (<.001) |
| **Has_Diabetes (Yes)** | 0.79 (0.023) | 0.79 (0.084) | 0.82 (0.109) | 0.64 (0.313) | 0.07 (0.917) |
| **Has_High_Cholesterol (Yes)** | -0.22 (0.400) | -0.16 (0.644) | 0.19 (0.621) | 0.15 (0.743) | 0.67 (0.200) |
| **Has_High_Blood_Pressure (Yes)** | 0.25 (0.230) | 0.67 (0.016) | 0.31 (0.309) | 0.66 (0.080) | 0.11 (0.800) |
| **BMI: Obese** | 6.64 (0.001) | 9.13 (<.001) | 8.90 (<.001) | 9.79 (<.001) | 10.70 (<.001) |
| **BMI: Overweight** | 4.97 (0.010) | 6.48 (<.001) | 5.33 (<.001) | 5.77 (<.001) | 5.65 (<.001) |
| **Age (per year)** | -0.01 (0.121) | -0.01 (0.206) | 0.00 (0.671) | 0.01 (0.422) | 0.01 (0.574) |
| **Weight_Baseline (per kg)** | -0.01 (0.059) | -0.03 (<.001) | -0.05 (<.001) | -0.07 (<.001) | -0.10 (<.001) |
| **R² / Adj. R²** | 0.067 / 0.061 | 0.115 / 0.110 | 0.181 / 0.177 | 0.214 / 0.209 | 0.262 / 0.257 |
| **F-statistic (P-Value)** | 12.66 (<.001) | 23.14 (<.001) | 43.99 (<.001) | 43.88 (<.001) | 53.21 (<.001) |
