# Supplementary material for: Impact of Digital Engagement on Weight Loss Outcomes in Obesity Management Among Individuals Using GLP-1 and Dual GLP-1/GIP Receptor Agonist Therapy: Retrospective Cohort Service Evaluation Study
Source: J Med Internet Res. 2025 Mar 31;27:e69466. doi: 10.2196/69466 (PMC11997532; doi:10.2196/69466)
Supplement: Multimedia Appendix 2 [file jmir_v27i1e69466_app2.docx]

**Table S3.** The table provides a detailed summary of weight change data across six time points (Month Rank 0 to 5) for three groups: All participants, those on Semaglutide, and those on Mounjaro. *P-value derived from independent t-test.

| Month Rank | Avg Weight Change (%) - All (95% CI) | Avg Weight Change (%) - Semaglutide (95% CI) | Avg Weight Change (%) - Tirzepatide (95% CI) | *P-Value** (Semaglutide vs Tirzepatide) |
| --- | --- | --- | --- | --- |
| 0 | 0 (0–0) | 0 (0–0) | 0 (0–0) | N/A |
| 1 | -3.17 (-3.23–3.11) | -2.54 (-2.66–2.42) | -3.54 (-3.60–3.47) | <.001 |
| 2 | -5.98 (-6.07–5.89) | -4.74 (-4.89–4.58) | -6.89 (-6.99–6.78) | <.001 |
| 3 | -8.31 (-8.43–8.18) | -6.96 (-7.14–6.78) | -9.63 (-9.78–9.48) | <.001 |
| 4 | -9.84 (-10.05–9.64) | -8.14 (-8.41–7.88) | -12.08 (-12.36–11.79) | <.001 |
| 5 | -10.74  (-10.96–10.51) | -9.46 (-9.71–9.21) | -13.88 (-14.29–13.48) | <.001 |
